# Perilipin-1 immunostaining improves semi-automated digital quantitation of bone marrow adipocytes in histological bone sections

**DOI:** 10.1080/21623945.2023.2252711

**Published:** 2023-08-30

**Authors:** Nicko Widjaja, Niki Jalava, Yimeng Chen, Kaisa K. Ivaska

**Affiliations:** Institute of Biomedicine, University of Turku, Turku, Finland

**Keywords:** Bone marrow adipocytes, image analysis, adiposity, adipocyte quantitation, perilipin-1

## Abstract

Bone marrow adipocytes (BMAds) are not just passive fillers inside the bone marrow compartment but respond to various metabolic changes. Assessment of those responses requires evaluation of the number of BMAds and their morphology for which laborious and error-prone manual histological analysis remains the most widely used method. Here, we report an alternative image analysis strategy to semi-automatically quantitate and analyse the morphology of BMAds in histological bone sections. Decalcified, formalin-fixed paraffin-embedded histological sections of long bones of Sprague-Dawley rats were stained with either haematoxylin and eosin (HE) or by immunofluorescent staining for adipocyte-specific protein perilipin-1 (PLIN1). ImageJ-based commands were constructed to detect BMAds sized 200 µm^2^ or larger from standardized 1 mm^2^ analysis regions by either classifying the background colour (HE) or the positive and circular PLIN1 fluorescent signal. Semi-automated quantitation strongly correlated with independent, single-blinded manual counts regardless of the staining method (HE-based: *r*=0.85, *p*<0.001; PLIN1 based: *r*=0.95, *p*<0.001). The detection error was higher in HE-stained sections than in PLIN1-stained sections (14% versus 5%, respectively; *p*<0.001), which was due to false-positive detections of unstained adipocyte-like circular structures. In our dataset, the total adiposity area from standardised ROIs in PLIN-1-stained sections correlated with that in whole-bone sections (*r*=0.60, *p*=0.02).

## Introduction

Adult bone marrow (BM) is an adipocyte-rich environment in which bone marrow adipose tissue (BMAT) increases with age and body mass [1]. Bone marrow adipocytes (BMAds) were considered for a long time to be passive energy-storing fillers in the marrow cavity of the skeleton. However, accumulating evidence shows that BMAds participate in both local and systemic metabolic processes in the skeleton, underscoring their likely contribution to skeletal homoeostasis [2–5]. Increased marrow adiposity is associated with several metabolic disorders, such as obesity, type 2 diabetes and anorexia (reviewed in [6]). Unlike peripheral adipocytes, the anatomical location of BMAds deep inside the skeleton makes it difficult to study these cells. At present, the histological analysis of a bone specimen remains relevant and practical to uncover the complex relationships between bone and fat. The extent of bone marrow adiposity, including changes in cell number and morphology, is often considered as one of the major reporting parameters [7].

Imaging BMAds in rodents *ex vivo* includes the use of a contrast agent to distinguish adipocytes from surrounding tissue and is usually performed by staining the lipid compartment. Computed tomography (CT) imaging, central to the bone imaging modality, has been regarded as a major tool to image both mineralised and BMAT compartments simultaneously, with the help of fat-staining osmium tetroxide [8]. However, CT imaging provides information only at the tissue level, making it impossible to investigate morphological changes in adipocytes at the cellular level. Various developments have been made to overcome this limitation, including the use of advanced software [9], nano-CT [10] and magnetic resonance imaging-based techniques [11]. However, many of these improvements are not immediately accessible to most laboratories and require technical expertise. Therefore, the aim of this study was to develop and validate methodologies to image, quantitate and analyse the morphology of BMAds in standard formalin-fixed paraffin-embedded (FFPE) histological bone tissue sections.

Histological analyses remain the gold standard in assessing bone marrow adiposity. However, the manual approach in quantitating and measuring the size of individual BMAds is time-consuming and can be prone to user bias. Attempts to automate this process through digital pathology tools have progressed rapidly with more features available to extract quantitative information in histological images. In FFPE histological bone sections, delipidated BMAds classically known as ‘adipocyte ghosts’ are identified by tracking background-coloured circular/oval voids [12,13]. This detection strategy offers good reproducibility in tightly packed adipocyte clusters with intact cytoplasmic boundary commonly observed in peripheral adipose tissues [12,14–17]. However, the evaluation of bone marrow adiposity based on the unstained area is prone to errors related to false-positive detection of other similar structures, such as blood vessels and empty interstitium, sharing the same circular morphology and pixel classification as BMAds. Consequently, we hypothesised that a specific histological staining to identify adipocytes, such as an immunostaining for lipid droplet-associated protein perilipin-1 (PLIN1) [18], would improve the detection accuracy.

Here we report the development and validation of a simple, semi-automated workflow using an open-source image analysis software ImageJ to detect and quantitate BMAds in FFPE histological bone sections. BMAds are positively stained for PLIN1, which offers an alternative in the adipocyte detection guide on the basis of positive fluorescent signal tracking. This strategy lowers the detection error-rate in comparison to the standard background-coloured detection in HE-stained histological sections. In addition to the quantitation feature, our script can also perform morphological analysis to extract the size of individual adipocytes. In our analysis of 24-week-old homoeostatic male Sprague-Dawley rats, we identified morphological differences of BMAds in different regions of rodent tibia, which may suggest heterogeneity in adipocyte population in the marrow.

## Materials and methods

### Sample preparation

Femurs and tibias from 24-week-old male Sprague-Dawley (SD) rats were harvested, fixed overnight in 10% v/v neutral-buffered formalin/PBS solution and decalcified in decalcifying solution (10% EDTA, 1 M NaH_2_PO_4_, 0.5 M Na_2_HPO_4_) for 16 days. Decalcified bones were paraffin-embedded and sectioned for staining. In brief, bones were oriented longitudinally, trimmed to approximately 40% deep from the surface to expose the maximum extent of the BM cavity and sectioned as 4-µm-thick sections.

### Histological staining

Consecutively sectioned FFPE histological bone sections (4 μm) were stained with either standard haematoxylin and eosin (HE) or by immunofluorescent staining targeting lipid droplet-associated protein PLIN1. Briefly, slides were incubated at + 55°C to improve tissue adhesiveness for one hour. Slides were then rehydrated in a series of descending concentration of ethanol. Antigen was retrieved by heating the slides in Tris-EDTA buffer (10 mM Tris, 1 mM EDTA, 0.05% Tween-20, pH 9) with a domestic microwave appliance at 160 W for 14 min. Tissue sections were permeabilized with 0.1% v/v Triton X-100/Tris buffer (15 mM Tris-HCl, 137 mM NaCl, pH 7.6; TBS) for 10 min, then blocked with 10% v/v goat serum/TBS (Abcam, Cambridge, UK) at RT for 1 h. PLIN1 was stained with 2 μg/mL rabbit polyclonal anti-PLIN1 (Thermofisher, Massachusetts, USA; PA1–1051, AB_2167579) at + 4°C overnight. Sections were subsequently stained with 2 μg/mL Alexa Fluor® 594-conjugated goat polyclonal anti-rabbit IgG (Abcam, Cambridge, UK; ab150080, AB_2650602) in the dark at RT for 1 h. Antibodies were reconstituted in 1% v/v goat serum/TBS. All washes between steps were performed in 0.1% v/v Tween-20/TBS. Tissue autofluorescence was reduced with Vector TrueView Autofluorescence Quenching Kit (Vector Laboratories, California, US) according to the manufacturer’s protocol. Slides were coverslipped with Vectashield Vibrant with 0.9 μg/mL DAPI (Vector Laboratories, California, US) and cured overnight prior to imaging.

### Image acquisition and regions of interest

Imaging of stained histological sections was performed with the highest available lens configuration. HE-stained slides were imaged with a Pannoramic 1000 slide scanner (3DHISTECH, Budapest, Hungary) with the following setting: (1) Objective magnification 40X; (2) Multiple Z-stack scanning mode; (3) Linear colour calibration. PLIN1-stained slides were imaged with a Pannoramic Midi slide scanner (3DHISTECH, Budapest, Hungary) with the following setting: (1) Objective magnification 20X; (2) TRITC and DAPI filter set (Zeiss, Oberkochen, Germany) with the exposure time 1000 ms and 300 ms, respectively; (3) Digital gain index of 2 for the TRITC channel. A standardised imaging setup was maintained for each imaging session. For the analysis, excess background staining from immunofluorescent stained sections was removed by using an auto-fluorescent filtering feature in CaseViewer version 2.4 (3DHISTECH, Budapest, Hungary). For femurs, the regions for analysis were defined as 1 mm × 1 mm squares which were drawn 2 mm and 6 mm proximally from the distal femur, using the most proximal point of the growth plate (GP) as a reference point (Figure S1a). This resulted in the extraction of two regions: ROI-1 (2 mm, containing both BM and trabecular bone) and ROI-2 (6 mm, only BM). For tibias, similar 1 mm × 1 mm regions of interest were drawn 3 mm and 9 mm distally from the midpoint of the GP of proximal tibia to extract ROI-1 and ROI-2, respectively (Figure S1b). Further, an additional analysis region in the distal tibia enriched with BMAds was included in the analysis (ROI-3). Annotated regions were exported as TIF-format images for subsequent analyses. Two validation datasets consisting of 40 images (ROI-1 and ROI-2 from femur and tibia, *n* = 25; ROI-3 from tibia *n* = 15) per staining method were generated.

### Analysis of regions of interest

Two semi-automated image analysis workflows were developed in ImageJ version 1.53c [19] to quantitate BMAds from two image sets based on the staining method employed (*n* = 25 per set). The performance of the scripts was first evaluated by comparing the detection counts between semi-automated and manual quantitation which was counted independently by two trained observers blinded to the sample type and outcome, similar to the work by Zhi and colleagues [16]. Next, the morphology of BMAds was compared in consecutive sections (ROI-2, 8 µm apart in depth) between staining methods from four different datasets. A dataset is defined as six consecutive sections (4 µm apart) from one animal with alternating sections stained with either HE (section levels 1, 3 and 5) or PLIN1 (section levels 2, 4 and 6). Further, the total adipose tissue area in pre-defined 1 mm × 1 mm regions (Ad.Ar/mm^2^, %) estimated by our workflow was compared to that in the entire marrow cavity (Ad.Ar/Ma.Ar, %) estimated by semi-automated image analysis plugin MarrowQuant in QuPath version 0.1.4 [12] to validate whether the selected ROI represented bone marrow adiposity in the entire marrow cavity.

For HE-stained images, BMAds were detected and quantitated using the negative-detection method. Briefly, the image was segmented to separate the background representing adipocyte objects from the foreground. Segmented image was then morphologically processed to improve adipocyte morphology by applying an opening filter with a radius of 10 pixels. Incomplete objects were digitally connected by analysing the local maxima between objects by using an open-source adjustable watershed plugin (available at https://imagejdocu.list.lu/plugin/segmentation/adjustable_watershed/start). Every object between 200 and 4000 μm^2^ in size and a circularity value between 0.5 and 1.0 was detected and counted as a BMAd using the native particle analysis command. Incomplete objects at the edges of the image were excluded. This lowest size limit for the detection of adipocyte was based on previous reports on the size range of BMAds accounting for microvascular structures in the bone marrow [12,13].

For immunofluorescent-stained images, the image was first segmented to separate the fluorescent signal (foreground) from the background. Segmented image was de-speckled to remove non-specific signals with a size of 200 μm^2^ or less [13] using the native particle analysis function and reregistered as background pixels. Local maxima analysis was performed to connect incomplete objects using the adjustable watershed plugin. BMAds were detected and quantitated using identical parameters as in the previous workflow. Both HE-based (Script 1) and immunofluorescent-based (Script 2) scripts are available in the supplementary material section.

### Statistical analyses

Data are presented as means with a standard deviation or medians with an interquartile range (IQR). Quantitative data are reported as the number of adipocytes per region of interest (N.Ad/mm^2^) or as area of individual adipocytes (Ad.Ar, μm^2^). The detection error-rate of the ImageJ-based quantitation was defined as an absolute ratio (%) of count differences over the manual count. Student’s t-test was performed to compare mean detection errors between the methods. Mann-Whitney U-test was performed to compare median distribution of BMAd size in different anatomical locations of the tibia. Pearson correlation was evaluated to study the association between different methods of adipocyte quantitation (ImageJ versus manual count and ImageJ versus MarrowQuant). Two-way ANOVA with Sidak’s correction for multiple comparisons was performed to compare the mean frequency of size distribution of BMAds in different regions of the tibia. Sample size and detailed parameters are indicated in figure legends whenever applicable. Statistical analysis was performed with Prism version 8.4.2 (GraphPad, San Diego, USA). P-values of less than 0.05 were considered statistically significant.

## Results

### Detection of BMAds in stained histological bone sections

**Figure 1.**
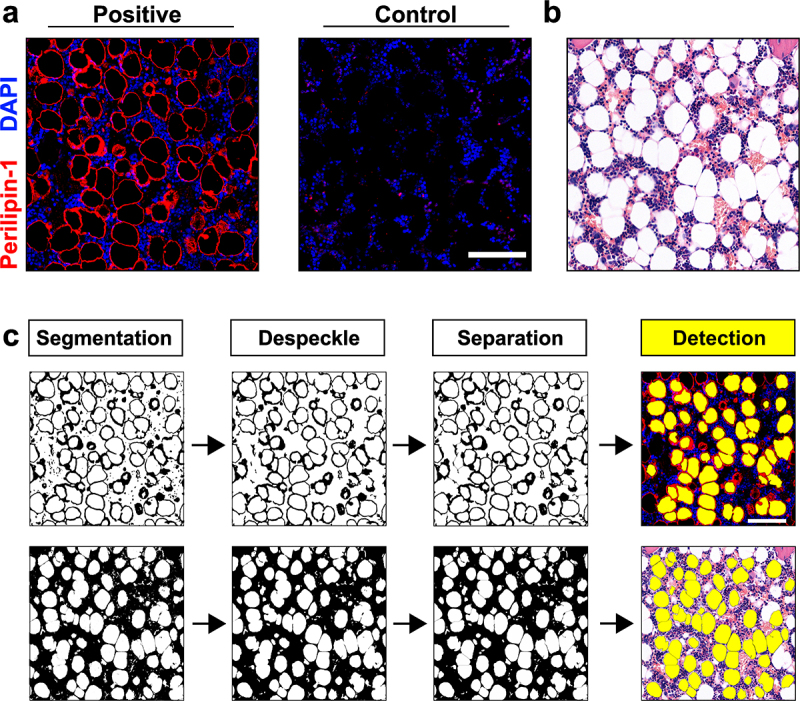
Semi-automated quantitation of bone marrow adipocytes in histological bone sections. (a) BMAds are positive for PLIN1. (b) In HE-stained sections, BMAds are identified as circular/oval empty regions known as ‘ghost adipocytes’. (c) The detection and quantitation script in (a) and (b) starts by segmenting, de-speckling and separating the adipocyte object from the images. The script detected 55 and 59 BMAds per field in PLIN1- and HE-stained samples shown in (c), respectively. Scale bar in (a) and (c): 100 μm.

BMAds were positive for PLIN1 and ring-like structures were observed within the adipocytes at the surface of the lipid droplet (Figure 1(a)). In HE-stained sections, BMAds were identified as circular/oval voids (adipocyte ghosts) with a thin cytoplasmic remnant (Figure 1(b)). Next, we developed two image analysis workflows to segment, de-speckle, separate and detect BMAds from PLIN1- or HE-stained input images (Figure 1(c)). Here, the user specifies the appropriate threshold value to segment adipocyte classifiers (a positive PLIN1 signal or a negative background pixel, respectively). The PLIN1 immunostaining was more specific for the detection of adipocytes as the vascular structures remained unstained (Figure S2a). However, image noise with the size range of less than 200 µm^2^ predominated the detection and was therefore excluded in the analysis (Figure S2b and c). Using the present quantitation parameters and the adipocyte size threshold of 200 µm^2^, the number of detected BMAds was on average 364 ± 56 and 243 ± 59 BMAds in PLIN1- and HE- stained 1 mm^2^ metaphyseal trabecular-enriched regions, respectively (ROI-1, *n* = 5). In marrow-rich diaphyseal regions (ROI-2, *n* = 20), the script quantitated on average 403 ± 48 and 409 ± 73 BMAds stained with PLIN1 or HE, respectively.

### PLIN1 immunostaining improves the detection of BMAds over HE-stained images

**Figure 2.**
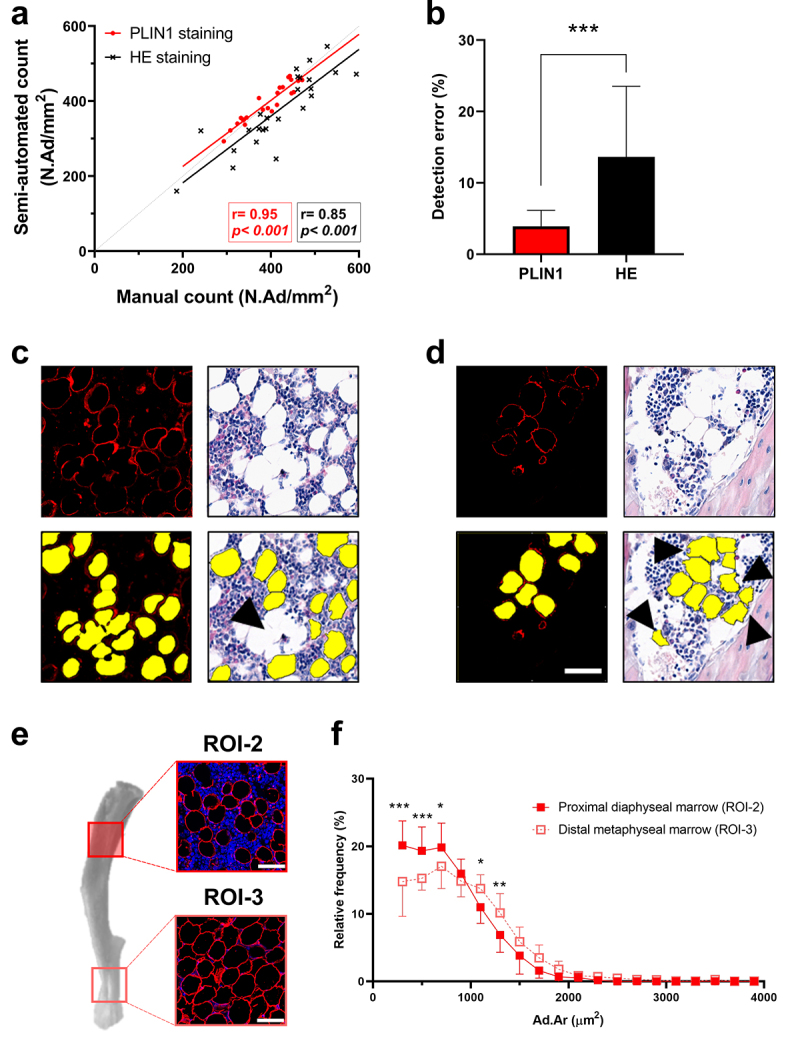
Perilipin-1 immunostaining improves the quantitation of bone marrow adipocytes in histological bone sections. (a) Both semi-automated quantitation methods correlate with the independent manual count. (b) Detection of PLIN-1 positive objects reduces the quantitation error-rate significantly (***p < 0.001 by Student’s t-test, *n* = 25 per staining method, ROI-1 *n* = 5 and ROI-2 *n* = 20) when compared to background-coloured object detection in HE-stained histological sections; this is due to better resolving capacity in (c) clustered objects and (d) reduction in false-positive detection (black arrowheads). Detected BMAds are represented with yellow overlay with a black line. (e) BMAds in the proximal tibia are enriched with bone marrow cells compared to those in the distal tibia. (f) Morphology analysis reveals significantly larger BMAds (*p* < 0.001, Mann-Whitney U-test) in distal metaphyseal marrow (ROI-3) compared to proximal diaphyseal marrow (ROI-2). Biological replicate *n* = 6 rats, and three intact contiguous sections were analysed for each rat (depth-distance between sections 8 µm). Each bin represents the analysis of 15 images. Data are presented as the mean frequency of BMAds (%) in a specified size range with a bin width of 200 μm^2^ starting from a bin centre of 300 μm^2^ (200–400 μm^2^) up to 3900 μm^2^ (3800–4000 μm^2^). *p < 0.05, **p < 0.01, ***p < 0.001 by two-way ANOVA with Sidak’s correction for multiple comparisons. Scale bar in (d) and (e): 50 μm.

We then checked the performance of both quantitation methods by comparing semi-automated counts with the independent and blinded manual counts. From the two validation datasets (ROI-1 and ROI-2, *n* = 25), we found a strong linear correlation between semi-automated and manual quantitation of BMAds in PLIN1-stained (*r* = 0.95; *p* < 0.001) and HE-stained (*r* = 0.85, *p* < 0.001) histological bone sections (Figure 2(a)). Although both staining methods were generally acceptable for image analysis, we found that HE-stained sections had higher detection error rate compared to PLIN1-stained sections (14% versus 5%, respectively; *p* < 0.001; Figure 2(b)). Irresolvable, tightly packed clusters of BMAds contributed to this error, increasing the likelihood for the underestimation of the number of BMAds detected in HE-stained sections (Figure 2(c)). False-positive detections appeared to be more common in HE-stained sections than in PLIN1-stained sections, especially in regions with circular interstitial spaces (Figure 2(d)). We further evaluated whether the adiposity in the selected 1 mm^2^ marrow-rich region (ROI-2) correlated to the adiposity in the entire marrow area in the histological tissue section, estimated from HE-stained sections by MarrowQuant. This fraction of marrow area selected at a pre-specified distance moderately correlated to the whole bone marrow adiposity (*r* = 0.60; *p* = 0.02) as shown in Figure S1c. There was no significant difference in the total adipose tissue area between pre-defined analysis regions (Ad.Ar/mm^2^, %; 40.0 ± 6.0%) and the entire marrow cavity (Ad.Ar/Ma.Ar, %; 31.8 ± 4.2%; *p* = 0.54) (Figure S1d). The limitations of both scripts are summarised in Figure S3. In addition, we analysed contiguous sections from each staining method and found that our PLIN1-based quantitation was presented with less variation in the median size of BMAds (Figure S4a), corresponding to the variation in whole-section adiposity in four different datasets of homoeostatic SD rats (Figure S4b). There were no significant differences in the minimum (Figure S4c) or the maximum (Figure S4d) adipocyte size between the contiguous sections or the staining methods.

### BMAds were larger and less interspersed with bone marrow cells in the distal tibia

Morphological assessment of BMAds from different regions in the tibia suggested that BMAds in the proximal, diaphyseal marrow region (ROI-2) were enriched with bone marrow cells, whereas more tightly packed adipocytes with less marrow cells were observed in the distal metaphyseal marrow region (ROI-3; Figure 2(e)). Size distribution of BMAds in ROI-2 was analysed in three contiguous sections (8 µm distance between sections) and compared to those in ROI-3. BMAds were significantly smaller in size (*p* < 0.001) in ROI-2 (median 698 µm^2^, IQR 455–991 µm^2^) compared to those in ROI-3 (845 µm^2^, 541–1199 µm^2^; Figure 2(f)).

## Discussion

In this study, we report a systematic comparison of quantitation and morphological analysis of BMAds in FFPE histological bone sections stained with either HE or adipocyte-specific protein PLIN1. The semi-automated workflows developed in this study significantly correlated with the respective manual cell counts and provided additional information on the morphology of BMAds. Additionally, PLIN1 immunostaining has been previously reported in extramedullary adipocytes [20,21], *in vitro* differentiated adipocytes [22] and bone marrow tissue sections [23–25]. Therefore, we suggest positive fluorescent signal tracking as a strategy that detects BMAds more accurately when compared with the commonly employed background-colour tracking method [12,15,16].

The detection of BMAds in HE-stained bone sections often presented with false-positive detections due to the following reasons. First, non-adipocyte circular structures such as blood vessels or interstitial spaces were incorrectly detected as BMAds due to the detection strategy in tracking all background-coloured pixels (Figure S3a and d). Second, the segmented erythrocytes or other small stained objects on larger blood vessels or empty areas caused the watershed algorithm to fragment that area producing small multiple objects that fall within the size and shape detection parameter (Figure S3b and e); these are common errors reported in many analyses of BM cellularity [12,26,27]. We added an adjustable watershed command allowing the user to set a higher tolerance value to minimise this hyper-fragmentation issue, although it may compromise object separation in regions with tightly clustered BMAds, underestimating the final count in general. Alternatively, the user may also exclude vascular or other unstained regions using the ImageJ built-in wand tracing tool. However, manual exclusion would defeat the purpose of automation and may introduce user bias in a large-scale analysis.

The detection strategy in PLIN1-stained sections (Script 2) was able to correct both errors described above, resulting in a significant reduction of the detection error-rate compared to the HE-based Script 1. Taking specific fluorescent signal as a detection guide helps to ignore many similar competing features that are not stained, especially in regions enriched with circular microvascular structures [28]. This translates to less false-positive detections in BMAd-sparse regions. However, as with many other bioimage-based image analysis workflows, it is important to note that the quality of staining dictates the detection performance [16]. Poor quality such as a high staining background [29–31] would render the algorithm non-specific and reduce the detection performance by introducing false-positive detections similar to the error in Script 1 (Figure S3g and j). Further, the alteration of cellular morphology due to sample processing may affect the morphological analysis in that incomplete cellular boundary would underestimate the area (Figure S3h and k). Residual detection antibodies may also disrupt the algorithm by introducing dot-like features for the watershed to fragment the area. We have added a module to reduce this error by removing non-specific small objects, although the extent of that feature may be overpowered by larger residual objects. Nevertheless, a successful and specific immunofluorescent staining opens more possibilities for multispectral analyses. For instance, the work by Scheller and colleagues [32] in addressing the subpopulation of BMAds has promoted the research to delineate the heterogeneity of BMAd populations using immunofluorescent-based methods [23,24].

We acknowledge that there are limitations in our approach. First, we focused mainly on the specific adipocyte detection thereby ignoring other BM cell populations. This may limit the clinical use in simultaneously assessing both haematopoietic and adipocytic compartments of the BM [13]. In addition, only unilocular BMAds with a pre-defined size range between 200 and 4000 μm^2^ were analysed with our scripts, thereby omitting the detection of very small adipocytes (<200 μm^2^). This omission overcomes the predominant false detection of PLIN1-positive objects that are morphologically distinct from mature adipocytes, including secondary detection-antibody complex and circular digital bridges outlining the space between adipocytes. The analysis of small adipocytes may be useful for the evaluation of *de novo* adipogenesis or the browning of adipocytes [21,22,33,34], which might have been missed in this study (Figure S3c, f, i and l). However, we assume that such evaluation may be difficult to capture at the histological level with different analysis planes that do not reflect the complete three-dimensional morphology. Our results consistently showed a regional trend of larger sized BMAds in the distal tibia compared to the proximal tibia, similar to the work reported by Scheller and colleagues [32], implying the need for a standardised analysis. The size of BMAds can vary greatly among species and skeletal locations. For instance, BMAds appear to be, on average, larger in size in humans [35] and rabbits [36], compared to rodents [12,32]. For clarity, our suggested size range covers sizes of BMAds typical to that of about 24-week-old Sprague-Dawley rats [33]. We therefore recommend the user to adjust the range depending on the research design.

Second, we acknowledge that we were unable to expand the analysis regions to the magnitude of whole-section adiposity as reported elsewhere [12,13,27,37]. However, we hosted our script in popular image analysis software ImageJ for better reachability when processing an extensive range of image file formats. Indeed, the percent adiposity in our dataset is in line with that in the whole-section dataset, as suggested in the guidelines for analysing bone marrow adiposity [7,38]. However, we acknowledge that our analysis regions may differ in animal models other than 24-week-old homoeostatic male Sprague-Dawley rats. The uneven distribution and expansion pattern of BMAds in the marrow cavity make it important to address this issue as randomly selected analysis regions may present a varying degree of adiposity [12,32]. Therefore, selecting pre-defined analysis regions systematically would minimise selection bias, especially in the assessment of adiposity with interventions. We thus recommend the user to systematically compare the fraction of their selected analysis regions to whole-marrow adiposity acquired from other analysis tools such as MarrowQuant [12,13].

Finally, it is still unclear whether the method is sensitive enough to detect changes in the adipocyte size in response to various pathophysiological challenges, which were not performed in this study. However, we observed less morphological variability between sections stained with PLIN1 compared to HE when analysing homoeostatic datasets (Figure S4). It is advisable to evaluate the analysis tool in determining whether variation in the data originates from a true biological response or the technical limitation discussed above.

In summary, here we report an alternative workflow for the digital detection and quantitation of BMAds in FFPE histological bone sections on the basis of specific and positive PLIN1 signal detection. This reduces false-positive detections of adipocyte-like structures such as microvasculature and empty interstitium that are common in background-coloured object tracking as demonstrated in the HE-based quantitation workflow. Further, our image analysis workflow has the capacity to perform morphological analysis which adds many quantitative aspects in the context of adipocyte size (e.g. area, diameter and perimeter) and shape (circularity and roundness), among others. Future direction in the software development for analysing larger fluorescent-based analysis regions would expand this detection strategy to fully quantitate whole bone marrow adiposity in histological bone sections.

## Supplementary Material

Supplemental Material

## Data Availability

The workflow presented in the supplementary material can process and analyse any images containing stained adipocytes. Original data is available upon request.
