# Supplementary material for: Perilipin-1 immunostaining improves semi-automated digital quantitation of bone marrow adipocytes in histological bone sections
Source: Adipocyte. 2023 Aug 30;12(1):2252711. doi: 10.1080/21623945.2023.2252711 (PMC10472850; doi:10.1080/21623945.2023.2252711)
Supplement: Supplemental Material [file KADI_A_2252711_SM2136.zip › Supplementary_Figures.pdf]

Supplementary Figures

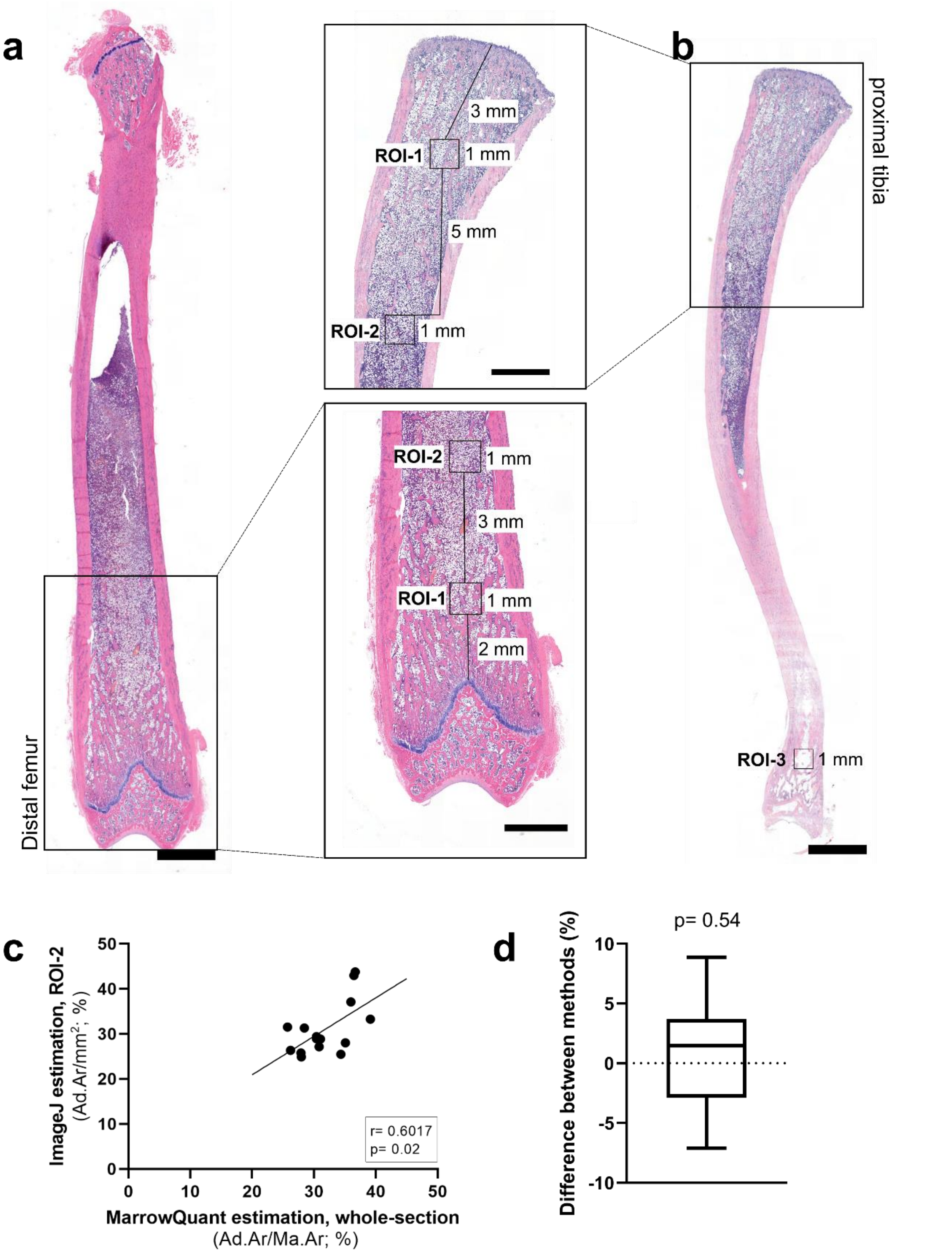

Figure S1. Analysis of pre-defined regions of interest represents whole-marrow adiposity. The regions for bone marrow adiposity analysis were extracted from (a) distal femur and (b) proximal tibia as 1 mm x 1mm square marrow regions containing both marrow and trabecular

bone (ROI-1), marrow only (ROI-2) and BMAds-enriched distal tibia (ROI-3). (c) Analysis of the unbiased selection of a region at a pre-specified distance (ROI-2) correlates to the whole bone marrow adiposity evaluated by MarrowQuant (r= 0.6017, p= 0.02; n= 15). (d) There was no statistically significant difference in the mean total adipose area between the whole marrow cavity and the predefined ROI-2 (p= 0.54 by one-sample t-test to test whether the mean difference was 0%) evaluated by MarrowQuant and PLIN1-based quantitation method, respectively. Scale bars: 3 mm in (a) and (b), 2 mm in each insert. Note that the epiphysis and part of growth plate (GP) in (b) was detached during sample processing.

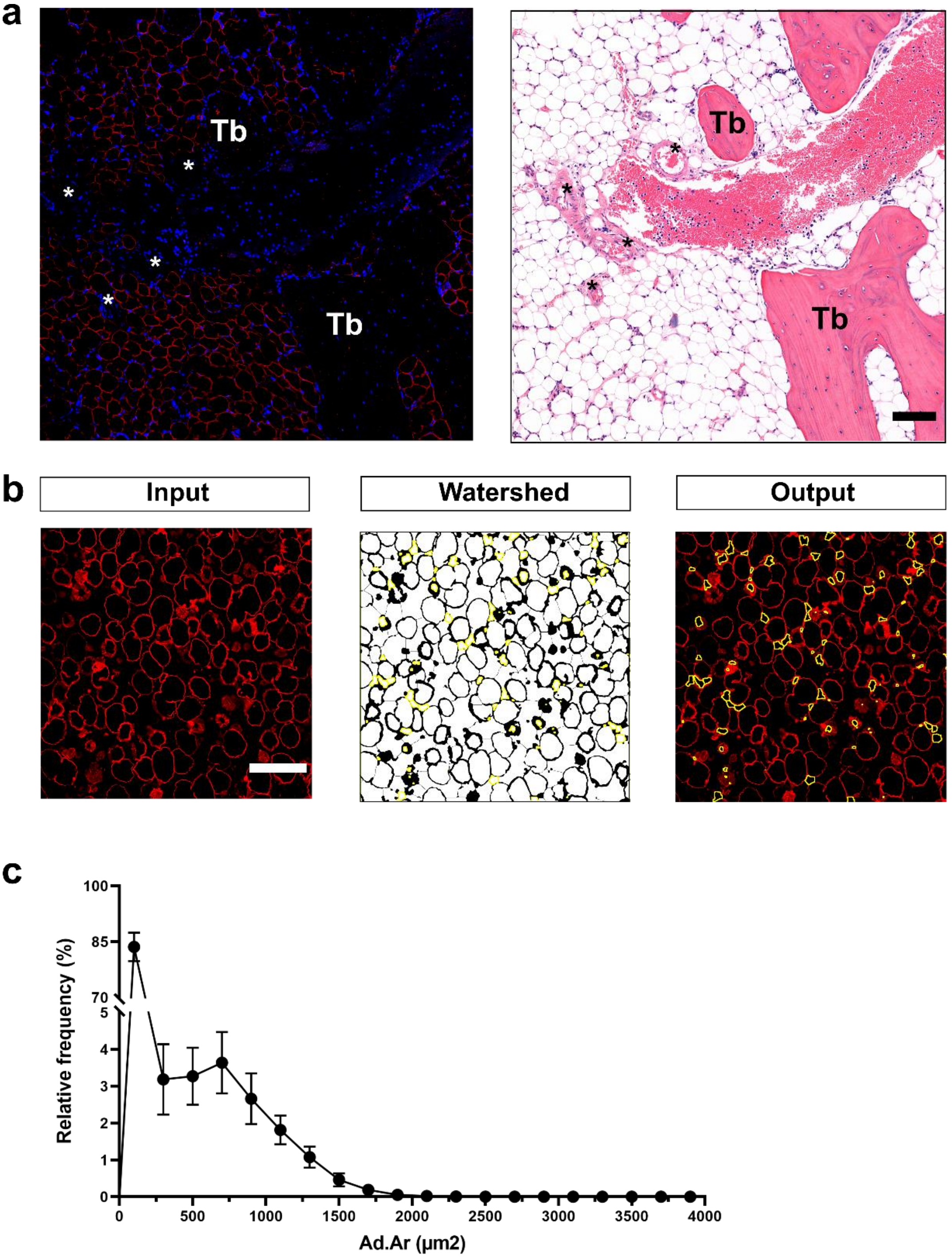

Figure S2. The detection guide for PLIN1-positive structures in histological bone sections. (a) PLIN1-stained objects are specific for BMAds. Bone marrow vasculature (*) was devoid of PLIN1 and therefore not detected as BMAds. (b) The inclusion of BMAds sized < 200 μm^2 revealed multiple false-positive non-adipocyte structures originating from secondary antibody complex and digital bridge connection of the watershed feature. (c) These artefacts masked the

detection of small adipocytes <200 μm^2 and contributed to the relative frequency of detected BMAds. Tb: Trabecular bone. Scale bar in (a) and (b): 100 µm.

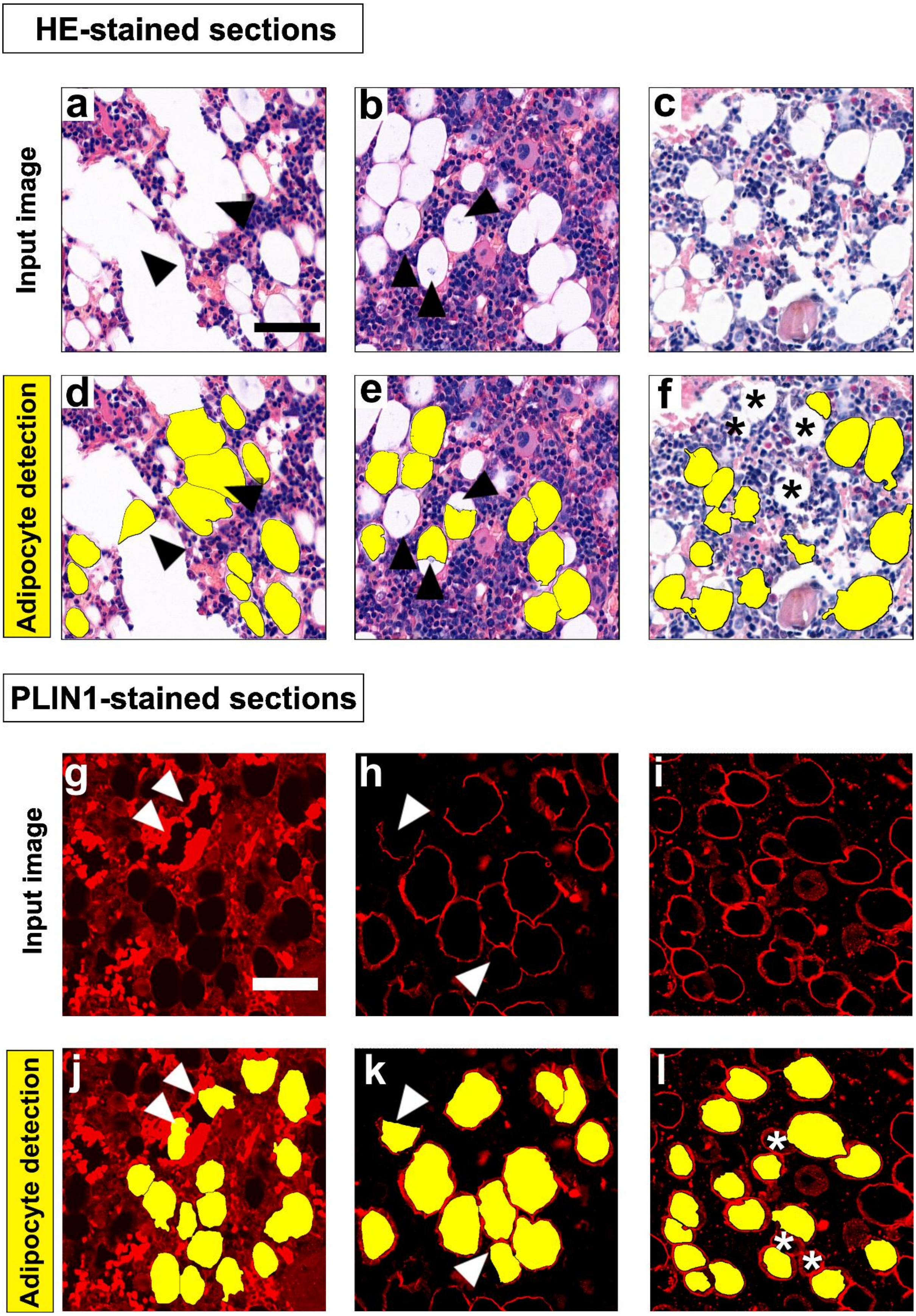

HE-stained sections
Input image
Adipocyte detection
a
b
c
d
e
f
PLIN1-stained sections
Input image
Adipocyte detection
g
h
i
j
k
l

Figure S3. Limitations of quantitation scripts. (a,d) In HE-stained sections, the workflow (Script 1) will always take background-coloured objects as adipocytes, producing a false-positive detection. (b,e) A staining artefact on adipocytes disturbs the watershed process, resulting in misdetection or object fragmentation (black arrowheads). (g,j) In PLIN1-stained sections, highly auto-fluorescent sections invalidate the script processing logic (Script 2) by classifying empty vascular region as adipocytes (white arrowheads) and should not be analysed with the script. (h,k) An incomplete fluorescent signal due to morphological alteration during the sample processing steps underestimated the area detected by the workflow (white arrowheads). (c,f,i,l) Regardless of the staining method, the user must pre-specify the adipocyte size detection. Here, as an example, we did not consider adipocytes < 200 μm^2 in the analysis, excluding small adipocytes (asterisks). Scale bar in (a) and (g): 50 μm.

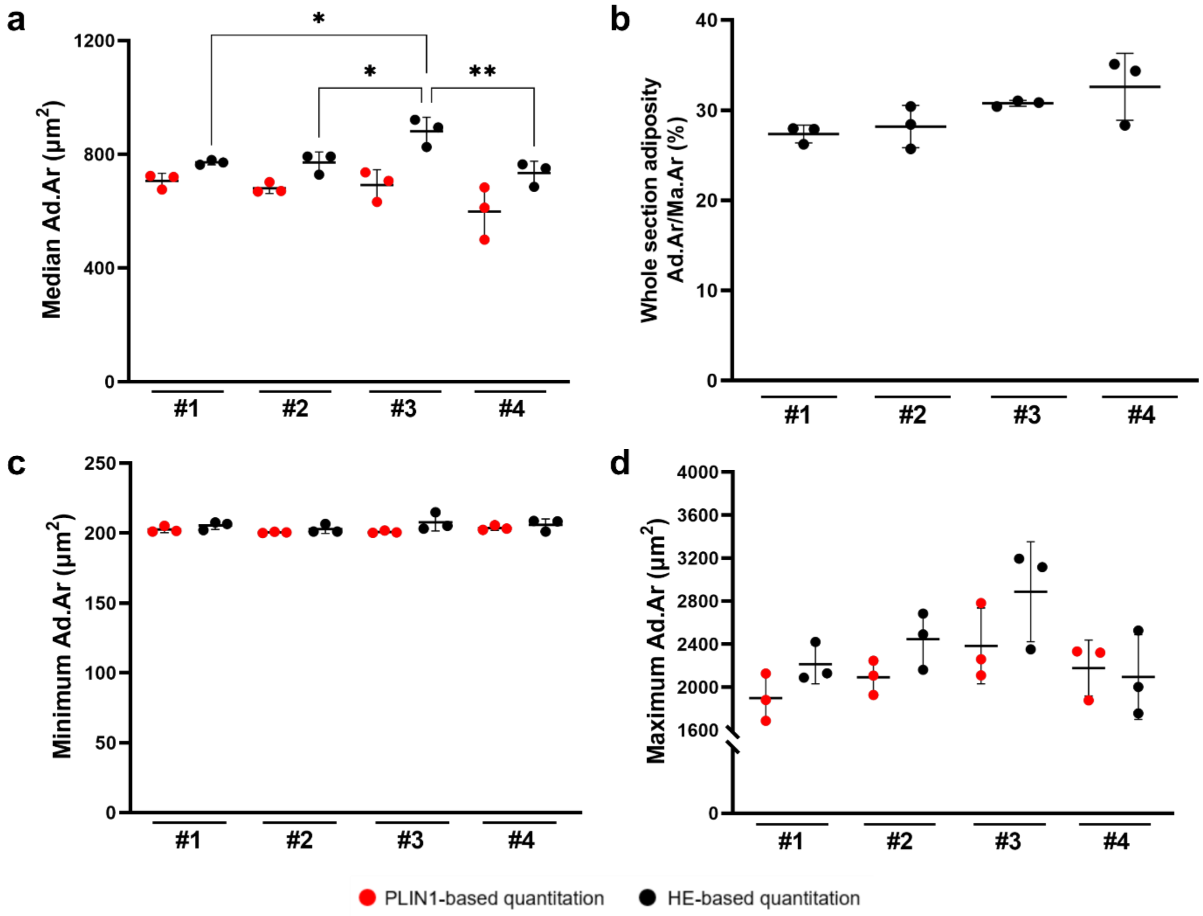

Figure S4. Morphological analysis of BMAds in consecutive HE-stained sections has greater variation compared to consecutive PLIN1-stained sections. (a,b) Comparison of the median area of BMAds in consecutive sections of PLIN1- (red circles) and HE-stained (black circles) sections. There is a significant difference between the datasets in HE-stained sections of 24-week-old homeostatic male Sprague-Dawley rats despite the similar whole-section adiposity estimated by MarrowQuant. (c) Minimum and (d) maximum size of BMAd detected by our scripts. Datasets (n= 4, three sections per dataset) in each staining method were compared by One-way ANOVA with Tukey's correction for multiple comparisons. *$p < 0.05$, **$p < 0.01$.
