# Supplementary material for: Perilipin-1 immunostaining improves semi-automated digital quantitation of bone marrow adipocytes in histological bone sections
Source: Adipocyte. 2023 Aug 30;12(1):2252711. doi: 10.1080/21623945.2023.2252711 (PMC10472850; doi:10.1080/21623945.2023.2252711)
Supplement: Supplemental Material [file KADI_A_2252711_SM2136.zip › Supplementary_Materials.docx]

**Supplementary Materials**

In this study (Widjaja et al. 2023, *Adipocyte* <http://dx.doi.org/10.1080/21623945.2023.2252711>), we developed two ImageJ-based scripts to quantitate bone marrow adipocytes in FFPE histological sections based on two staining methods: haematoxylin and eosin- and perilipin-1 –stained sections. To use the script, the user has to navigate to the following path (on Image J version 1.53c): Plugins > New > Macro. In a new window, the user has to select the IJ1Macro language from the menu bar and import the command lists. We embedded notes presented with double slash in some steps for clarity. The user will then open an image and run the script either individually or in batch mode. The latter requires additional commands, which are not included in this script for simplicity. The processing performance depends mainly on the user’s computer memory configuration. We have only tested our script to analyse 1 mm^2^ regions with a typical resolution of 10 MP.

The first script describes commands needed to quantitate adipocytes from haematoxylin and eosin stained sections. The segmentation process is more unified than that of immunostained images, as the script takes background colour located near the end of the histogram spectrum. Therefore, we chose a single threshold value applicable to other images. Both scripts require an additional plugin downloadable from:

<https://imagejdocu.list.lu/plugin/segmentation/adjustable_watershed/start>.

The second script describes commands needed to first segment PLIN1 signal from the background creating a ring-like structure as the principle for BMAd detection. An additional processing step is also added to despeckle small non-specific signal with the same intensity as PLIN1. This is essential for the watershed not to hyper-fragment the image causing false-positive detections. Similar to Script 1, the user can adjust the detection parameter for subsequent analyses.

**Script 1 – Haematoxylin and Eosin –stained section**

//Section 1 – Image preprocessing

run("Colors...", "foreground=white background=black selection=yellow");

//The user has to specify the scaling system based on the imaging modality used//

run("Set Scale...", "distance=3.0769 known=1 unit=micron");

rename("input");

run("8-bit");

//Section 2 – Segmentation

//This section aims to separate background colour (delipidated region of the adipocyte) from the foreground//

setAutoThreshold("Default dark");

//run("Threshold...");

setThreshold(240, 255);

//setThreshold(240, 255);

setOption("BlackBackground", false);

run("Convert to Mask");

run("Invert");

//Section 3 - Morphological operation (opening)

//This section aims to improve object (adipocytes morphology) and minimise false-positive detection around marrow vasculature by expanding the pixel of haematopoietic cells inside the vessel//

run("Options...", "iterations=5 count=4 black do=open");

//The user has to install the adjustable watershed feature to proceed. We provided the direct source for the installation above and in the original article//

run("Adjustable Watershed", "tolerance=5");

//Section 4 – Object detection

//This section aims to detect object based on the parameters set below//

//The user has to specify the detection parameter, including the size and circularity. Natively, the script will not detect incomplete object around the edge of the image. The user can ignore this feature by deleting the word “exclude” from the parameter below//

run("Analyze Particles...", "size=200-4000 circularity=0.5-1.00 display exclude clear summarize add");

//Section 5 – Data extraction

//This section aims to copy results directly to the clipboard allowing the user to directly import the data into a spreadsheet program//

String.copyResults();

//END OF THE SCRIPT//

**Script 2 – Perilipin-1 stained sections**

//Section 1 – Image preprocessing

//This section aims to equalise histogram from differently acquired images//

run("Colors...", "foreground=white background=black selection=yellow");

//The user has to specify the scaling system based on the imaging modality used//

run("Set Scale...", "distance=3.0769 known=1 unit=micron");

rename("input");

//For initial runs, the user can opt to duplicate the renamed image. By the end of the run, the original image remains active, in which the user can easily evaluate the detection performance. In subsequent run or with batch analysis, the command can be ignored by deletion//

run("Duplicate...", " ");

run("Bandpass Filter...", "filter_large=85 filter_small=1 suppress=None tolerance=5 autoscale saturate");

run("Median...", "radius=5");

//Section 2 – Segmentation

//This section aims to separate positive object from the background//

run("Threshold...");

//The user has to select the threshold value needed for segmentation. We made an interactive and live segmentation method for this purpose//

title = 'Set threshold value';

message = 'Adjust threshold value to segment image. \nStandardise segmentation method by selecting value close to the histogram peak \nClick "APPLY" on the threshold dialog box \n \nClick OK to continue';

waitForUser(title, message);

setOption("BlackBackground", true);

run("Convert to Mask");

run("Invert");

//Section 3 - Morphological operation (despeckling)

//This section aims to improve object (adipocytes morphology) by eliminating non-specific signal such as autofluorescence or residual detection antibody complex//

run("Invert");

run("Analyze Particles...", "size=0-200 circularity=0.1-1.00 show=Masks display clear summarize add");

run("Colors...", "foreground=black background=white selection=yellow");

selectWindow("input-1");

n = roiManager("count");

for (i=0; i<n; i++) {

roiManager("select", i);

roiManager("fill");

}

run("Invert");

//The user has to install the adjustable watershed feature to proceed. We provided the direct source for the installation above and in the original article//

run("Adjustable Watershed", "tolerance=5");

//Section 4 – Object detection

//This section aims to detect object based on the parameters set below//

//The user has to specify the detection parameter, including the size and circularity. Natively, the script will not detect incomplete object around the edge of the image. The user can ignore this feature by deleting the word “exclude” from the parameter below//

run("Analyze Particles...", "size=200-4000 circularity=0.5-1.00 display exclude clear summarize add");

//Section 5 – Data extraction

//This section aims to copy results directly to the clipboard allowing the user to directly import the data into a spreadsheet program//

String.copyResults();

//END OF THE SCRIPT//
